# Psychological Problems in the Context of Political Violence in Afghan Children

**DOI:** 10.1007/s11920-024-01496-2

**Published:** 2024-04-02

**Authors:** Laura Jobson, Daniel McAvoy, Sayed Jafar Ahmadi

**Affiliations:** 1 Turner Institute for Brain and Mental Health and School of Psychological Sciences, Monash University, Clayton, VIC 3800 Australia; 2 Centre for Humanitarian Leadership, Deakin University, Melbourne, VIC Australia; 3 Psychology Faculty, Bard College, New York, NY USA

**Keywords:** Trauma, Violence, Children, Afghanistan, Adolescent, Youth

## Abstract

**Purpose of Review:**

This review provides an overview of recent literature examining psychological problems in the context of political violence among Afghan children.

**Recent Findings:**

Using recent literature (2018–2023) we identified: 1) heightened levels of psychological problems experienced by children in Afghanistan; 2) the factors associated with these psychological problems, including loss of family and community members, poverty, continuous risk of injury and death, gender, substance use, war, daily stressors, and poor access to education; 3) psychological problems have potentially worsened since the 2021 political changes; 4) conflict and poverty have resulted in violence against children being a serious issue; 5) emerging psychological interventions have been adapted to Afghan contexts; and 6) there is a desperate need for psychological assistance and further research in the region.

**Summary:**

All children in Afghanistan have experienced conflict and political violence. While children are not responsible for this conflict, it has impacted their mental health. Further research is needed to examine the development and evaluation of interventions.

## Introduction

Afghanistan has endured over four decades of political violence, poverty and social injustice [1, 2] and in 2023 was ranked the least peaceful country in the world for the eighth consecutive year [3]. Generations of Afghans, and indeed every child presently in Afghanistan, have experienced the impacts of war and conflict [4•, 5]. Children – who make up more than half of the population in Afghanistan [4•] – are not responsible for political conflict and war, but generally suffer the most [6, 7]. Conflict and violence have had significant impacts on the mental health of Afghan children [2, 5, 8, 9]. Compounding matters, in August 2021, Western forces withdrew and the world watched on as the Taliban regained control of Afghanistan. The media and the United Nations were quick to report that “The needs of children of Afghanistan have never been greater” [10]; “Children and adolescents are struggling with anxieties and fears, in desperate need of mental health support” [11] and “Afghan children are growing up in deprived environments… [and] are in grave need of mental health support” [12]. Thus, questions rapidly arose regarding the current mental health of Afghan children.

Since 2021, the humanitarian situation in Afghanistan has worsened [3] and many questions related to the current mental health of Afghan children remain. Conflict related deaths have declined [3]. However, psychological impacts on the mental health of Afghan children in the context of endemic political violence remain significant. Empirically addressing these questions is an imperative as child mental health has been identified as a humanitarian priority [13–15] and the needs of children in humanitarian settings continue to receive insufficient attention in the field of psychology and psychiatry [16]. This review therefore aimed to provide a current overview of recent literature examining psychological problems, in the context of political violence, in Afghan children. We defined children and adolescents as those aged 0–24 years [17]. First, we outline the main psychological problems experienced by Afghan children and adolescents. Second, we provide a review of the recent literature examining these psychological problems. Third, we offer personal observations, based on our work. Finally, we provide a conclusion with recommendations.

## Main Psychological Problems Experienced by Children in Afghanistan

A significant proportion of the Afghan population experience posttraumatic stress disorder (PTSD), depression and anxiety [2]. Conflict and insecurity continue to have negative impacts on children’s mental health in Afghanistan [2, 6, 7]. While the World Health Organization notes adolescents globally may encounter mental health disorders at a rate of 13% [18], research continues to indicate poor mental health and heightened psychological distress among Afghan children [19]. In one of the earliest investigations, Panter-Brick and colleagues [8] found that two thirds of Afghan children reported frequently experiencing trauma and among their sample 22·2% met the criteria for a probable psychiatric disorder, 18.0% met criteria for emotional concerns, 4.8% met criteria for conduct problems, and 23.9% met criteria for PTSD. Additionally, psychiatric problems were associated with female gender and PTSD symptoms were associated with caregiver mental health, exposure to five or more traumatic events and child age [8]. More recent studies, have similarly demonstrated a significant proportion of Afghan youth are at substantial risk of psychiatric problems, PTSD, depression, and anxiety [19, 20•]. Thus, it is important to examine the current state of the literature relating to psychological problems in the context of political violence among Afghan children.

## Summary of Recent Literature Relating to Psychological Problems Among Afghan Children

While it was not the aim of this article to conduct a systematic review, we used several databases (Psycinfo, Medline) and specified search terms (‘Afghan’, ‘Afghanistan’ ‘child/ren’, ‘adolescent’, ‘youth’) to examine current literature assessing psychological problems experienced by Afghan children. We limited our search to articles published within the past five years (2018–2023) and focused on psychological problems of children living in Afghanistan. We identified six main areas of focus; 1) prevalence of psychological problems, 2) factors associated with psychological problems, 3) impacts of the Taliban, 4) violence against children, 5) psychological interventions adapted to Afghan culture and contexts, and 6) need for psychological support, assistance and interventions. These are discussed in turn below.

### Prevalence of Psychological Problems

Afghan children and adolescents are experiencing heightened levels of distress. Recent assessments suggest that over 30% of Afghan children have had exposure to psychological distress [4•]. Prevalence of single and multiple psychological trauma and distress among adolescents is around 28% [21]. A recent review, highlighted youth in particular were at greater risk for mental health problems and psychological distress [22•]. Children and adolescents are struggling with anxiety, fear, PTSD and depression [3, 23•, 24], which are causing severe negative outcomes [24]. Among a sample of school aged children (*N* = 2707) in eight regions of Afghanistan, a total of 52.75% of children were experiencing psychological difficulties, 39.19% emotional difficulties, 51.98% conduct challenges, and 15.37% hyperactivity/inattention [25]. Additionally, peer relationship problems were high and these psychological problems were impacting daily life [25]. Najm and colleagues [26], whilst establishing a child and adolescent mental health service in Herat, observed high prevalence of psychological disorders amongst those children and adolescents who presented to the clinic (*N* = 2448) over a three-year period. Epilepsy (34.0%) and intellectual disability (15.1%) were the two most common disorders, followed by depression (12.9%), and anxiety (12.5%). The least common disorders were psychotic disorder (1.8%), obsessive–compulsive disorder (1.5%) and speech disorders (0.9%). Some studies have highlighted post-traumatic growth (enhanced hope, spiritual wellbeing) achieved by Afghan adolescents over time [27]. Nevertheless, despite these findings, review articles have highlighted that evidence on prevalence rates of mental health disorders in Afghanistan is limited [28, 29].

### Factors Associated with Psychological Problems

Several factors have been associated with the heightened levels of psychological problems experienced by Afghan children and adolescents. These include loss of parent, family and community members (particularly if death was traumatic) [4•, 27], constant risk of death and injury, lack of social support [4•], decades of chronic socioeconomic health crises [24], being female [5, 21], substance use, bullying victimization, the experience of hunger, truancy [21] and exposure to terrorist attacks [25]. Additionally, being the first-born child of the family, daily stressors and greater war exposure have negative impacts on mental health [5]. Several studies focused on the impacts of gender. For instance, girls, when compared to boys, have been found to present with higher rates of anxiety, depression and daily stressors, while boys presented with greater rates of war experiences [5], conduct problems and total psychiatric difficulties [25].

School attendance has been associated with the mental health of Afghan children [25]. Among 2,707 school-aged children living in eight regions of Afghanistan, those who attended school were less likely to have emotional difficulties but were more likely to have problems with peer relationships [25]. Blum and colleagues found adolescents reported highly valuing education but believed boys to benefit more from education than girls [30]. Over 90% of parents reported that they expected their children to complete at least secondary education, regardless of the child's gender [30]. Trani and colleagues [31] examined whether an active education policy in Afghanistan promoting inclusion since 2005 had been effective. They found access to school and literacy did not improve between 2005 and 2013 for children and youth with disabilities (i.e., physical, sensory, mental), particularly for girls with disabilities. They also found evidence to indicate that school attendance might play a protective role for children with disabilities in Afghanistan. Thus, school attendance is important for children's mental health, across genders. Removing current restrictions on school attendance imposed since August 2021, particularly for girls, should be a priority in Afghanistan [25].

### Impacts of the 2021 Political Changes

Several studies specifically focused on the impacts of the 2021 political changes in Afghanistan on child and adolescent mental health. There have been additional challenges associated with these political changes. The majority of youth noted frustration with the security situation in Afghanistan [5]. The humanitarian crisis in Afghanistan worsened after the US and international allies withdrew [1]. Almost 60% of Afghans have been forced to flee their homes, children are facing life-threatening malnutrition, and many children have been separated from their families [23•]. Ahmadi and colleagues [20•] examined mental health among adolescents in the months following the changes in government in 2021. Of their sample (*N* = 376), 28.2% were at substantial risk for psychiatric problems and approximately half met criteria for probable PTSD, depression, or anxiety. Among girls, 47.5% were at considerable risk of having psychiatric problems (vs 14% of boys) and more girls (vs boys) met criteria for probable diagnosis of PTSD (79% vs 31%), depression (79% vs 26%), and anxiety (78% vs 21%). Younger age was associated with greater likelihood of probable psychiatric disorders [20•]. These statistics are generally higher than that observed in previous studies [8], indicating potential greater psychological problems among Afghan youth in recent years [20•].

### Violence Against Children

In the context of decades of poverty and conflict, violence against children in Afghanistan is a concern, with children being exposed to multiple forms of violence in the family, school and community [32–34]. Among a community sample (*N* = 145), 71% of children reported having experienced physical violence in the past year, with home being the most likely site in which the violence occurred, and physical violence being used as a means of discipline [33]. Community leaders, professional groups, and business owners in three Afghan districts (Kabul, Jalalabad and Torkham) perceived violence against children to be attributed to lack of education, poverty, and the impacts of war, with decades of poverty and armed conflict influencing how violence is recognized and perceived [34]. Corboz et al. [32] explored, among school children in Afghanistan, the prevalence of peer violence and associated factors. They recruited 770 children in 11 schools in Jawzjan province into a baseline study, which was part of an evaluation of a school-based peace education intervention. In their sample, 49.7% of boys and 43.3% of girls reported experiencing violence victimization at least once in the past month, and 17.6% of girls and 31.7% of boys reported perpetrating violence at least once in the past month, with significant overlaps between experiences of perpetration and victimization.

Peer violence was associated with food insecurity, child exposure to witnessing violence in the family, and child’s experience of corporal punishment at school and physical violence at home [32]. Li and colleagues [35] found a minority of adolescents endorsed violence, reported that specific circumstances justified violence (e.g., parents hitting children) and around a quarter approved of threatening a child if they spoke out against harmful traditional practices. Thus, while advocating for physical violence is socially unacceptable, under some conditions violence may be perceived by some as justified [35].

Child marriage is globally recognized as violating human rights and can impact mental health [24]. A study conducted in six Afghan provinces with low educational enrolment, found that, among their sample of 910 adolescents (12–15 years), both boys and girls believed marriage of girls under age 18-years increased risks of domestic violence [30]. Qamar and colleagues [36] used nationally representative data collected by the Demographic and Health Surveys to examine the relationship between family violence and child marriage in Afghanistan. They found in their sample (*N* = 21,324) that 15% of participants were married under the age of 15 and 35% were married between 15 and 17 years of age. After adjusting for age, place of residence, and socioeconomic status, the odds of sexual violence were 22% higher among women who married under 15 years of age compared to those were married as adults [24]. Dadras et al. [37] found in their study that over half of the Afghan women aged 15 to 49 years experienced intimate partner violence in the past year. Illiteracy and living in rural areas were associated with a higher risk and there was an elevated risk of mortality and morbidity among those under five years of age born to mothers exposed to intimate partner violence.

### Psychological Interventions adapted to Afghan Culture and Contexts

A small but expanding body of research is developing in the area of psychological interventions adapted for Afghan culture and contexts [22•]. A few recent studies have reported on the use of pharmacotherapy (i.e., anti-epileptics, anti-depressants, anti-psychotics, mood stabilizers) and psychological interventions (i.e., counseling, psychoeducation, cognitive-behavioral therapy, play therapy, group therapy, parenting interventions, speech therapy and applied behavior analysis) among children and adolescents in Afghanistan [26]. Corboz and colleagues [32] evaluated a school-based peace education and a community-based intervention to modify social norms and practices relating to violence in conflict resolution. They found that following the intervention there were significant reductions in violence at the school level (including peer violence perpetration, corporal punishment by teachers and peer violence victimization). Additionally, there were significant reductions in observations of family violence and experiences of corporal punishment at home, with a particularly strong effect found for girls. Both girls and boys had significantly more equitable attitudes towards gender and less violence-supportive attitudes relating to children's punishment, and significant reductions in symptoms of depression. Girls' school attendance also improved [32]. Their evaluation indicated the intervention has the potential to reduce various forms of violence [32].

Several interventions have targeted trauma and PTSD among Afghan adolescents. Ahmadi and colleagues [38] assessed the efficacy, feasibility and acceptability of modified written exposure therapy (m-WET) in treating PTSD symptoms among Afghan adolescent girls following a terrorist attack. Afghan adolescent girls were randomly assigned to m-WET (i.e., five daily group sessions whereby adolescents wrote about the terrorist attack), trauma-focused cognitive behaviour therapy (TF-CBT; an intensive five-session group psychological intervention), or a control group (which had no further contact). Acceptability and facilitator and participant satisfaction with m-WET was relatively high. At post-intervention, the m-WET group had significantly lower PTSD symptom severity compared to the control group and these gains were maintained at three-month follow-up. Moreover, there was no significant difference between the m-WET and TF-CBT groups. These findings indicate m-WET may have promise as a psychological intervention for PTSD among adolescent girls in Afghanistan [38].

Ahmadi and colleagues [39•] investigated the efficacy of Memory Training for Recovery–Adolescent (METRA) in improving mental health among adolescent girls in Kabul. The adolescents allocated to METRA received a 10-session group-intervention comprised of two modules (module 1: memory specificity training; module 2: trauma writing). Those in the treatment as usual (TAU) group received 10 adolescent health sessions. At post-intervention the METRA group had fewer symptoms of PTSD, depression, anxiety and psychiatric difficulties than those in the TAU group. These improvements continued to be observed at three-month follow-up. This trial was conducted in the months following the political changes in Afghanistan in 2021. Despite this presenting several difficulties for conducting the study as planned, METRA was able to be delivered to adolescents by facilitator with minimal training. Next, Ahmadi and colleagues [40] examined the feasibility, acceptability, and efficacy of METRA in improving psychological symptoms among Afghan adolescent boys in the aftermath of a terrorist attack. Again, they found that those offered METRA had significantly greater reductions in symptoms of PTSD, depression, anxiety, Afghan-cultural distress and psychiatric difficulties than did those in the control group. Additionally, those who completed METRA reported satisfaction with the intervention. However, it is worth noting that in this study, there were challenges in youth participation relating to security and competing education demands. Therefore, with some modifications, METRA appears a feasible psychological intervention for adolescents in Afghanistan [39•, 40].

### Desperate Need for Psychological Support, Assistance and Interventions

Finally, studies noted the desperate need for psychological support, assistance and interventions [4•, 41•]. There are very few studies investigating psychological interventions among Afghan children and adolescents, and the evidence produced is generally low quality [2]. Therefore, there is an urgent need for culturally appropriate innovative psychological interventions [2]. There is an urgent need for psychological training, the promotion of mental health services, public health awareness campaigns, and initiatives to assist re-connecting with family and loved ones [24], with a consideration of innovative systems-level approaches [42]. There is a need for immediate action plans from government and public health officials [24]. It is imperative that psychological research and the development of psychological interventions continues in Afghanistan, as the complexities of conducting research and delivering interventions reflect the reality of these contexts [39•, 40]. Afghan children and adolescents are needing, and deserving, of evidence-based psychological interventions, service delivery and psychological support [39•, 40].

## Personal Observations

In conducting the above review, it became apparent that few articles have focused on the mental health of children and adolescents in Afghanistan. This is problematic given the immense poverty, conflict and political violence experienced by children and adolescents in Afghanistan – potentially some of the highest rates experienced by children globally. Additionally, using our search terms, many of the studies focused on Afghan refugees resettled in high-income countries or the children of US veterans who had served in Afghanistan. This again highlighted a concerning gap in research; few studies have examined the prevalence of psychological problems, factors associated with these problems and the development and evaluation of psychological interventions for children and adolescents in Afghanistan. Again, this stands in contrast to the immense research on these topics among high-income countries – regions in which youth are experiencing immeasurably less poverty, war and political violence. This imbalance is exceptionally problematic and draws attention to the urgent need for greater research in this area to inform practice and policy.

Over the past few years our team has conducted several trials in Kabul, Afghanistan examining psychological interventions for adolescents with PTSD. In this work, we had a clearly structured, registered trial to be conducted in Kabul and Herat. However, just prior to our study commencing, the US and its allies withdrew from Afghanistan and the Taliban regained control. It is hard to articulate our feelings as we witnessed communities and research teams, whom we had worked closely with for decades, being at risk and the political, health and education systems quickly unravelling. We contemplated ceasing our work. However, based on discussions with community, we were urged to continue. Very few adolescents in Afghanistan receive mental health interventions due to limited health services, high costs, and a shortage of skilled mental health professionals [39•, 40]. Moreover, once the US and its allies withdrew, almost all mental health agencies ceased providing services in Afghanistan. Thus, to ensure some psychological interventions were being made available to youth, we continued our work. In continuing our work, we significantly shifted our design (e.g., shortening assessment sessions and treatment delivery). This highlighted to us the flexibility needed when conducting research and delivering an intervention in humanitarian contexts. We also ensured all interventions could be sustained and delivered beyond the life of the project.

Based on clinical observations, due to the prohibition of adolescent girls attending school by the Taliban and the restriction of mental health projects, it seems the psychological problems of adolescent girls have intensified. In individual and occasional group counseling sessions in early 2023, it was observed that girls deprived of education were experiencing high levels of depression, and suicidal thoughts were present. Reports on social media indicate suicide attempts, especially among women who have been liberated from Taliban abuse and torture. Many girls contemplating suicide have mentioned that, firstly, suicide is considered a grave and unforgivable sin in Islam, and secondly, they refrain from it to preserve the honour of their families.

Another concern expressed by adolescent girls is forced marriage. After the ban on attending school and the encouragement by the Taliban for early marriages, families are inclined to marry off their daughters at an early age. Parents, sometimes due to poverty or fear of the Taliban, are willing to arrange early marriages for their daughters. Another concern related to forced marriage and the prohibition of education is that it creates conditions for human trafficking, especially sex trafficking. In a study by Saramad and Ahmadi [43] on the areas and causes of trafficking of women and children, one of the most significant factors was forced marriage. When teenage girls are not allowed to go to school and are pressured by the.

Taliban and their families into early marriages, it becomes a conducive environment for human trafficking. The fear of forced marriage and the lack of education may trap girls in human trafficking; human trafficking option may offer an escape from forced marriage or perceived opportunities to continue education outside of Afghanistan.

## Conclusion

In conclusion, each child born and raised in Afghanistan has experienced impacts of political violence and conflict [5]. Children, while not responsible for political conflict and war, generally suffer the most [6, 7]. Conflict and violence have had significant negative impacts on the mental health of Afghan children. Prevalence studies indicate heightened psychological problems among Afghan children and adolescents, but also highlight a lack of research in this area. Factors associated with heightened levels of psychological problems include distress due to the persistent threat of injury and death, loss of family and community members, lack of social support, chronic socioeconomic health crises and poverty, gender, substance use, violence and war, daily stressors and poor access to education. There have been additional hardships following the changes in government in 2021, which have had further detrimental effects on mental health. In the context of decades of entrenched conflict, and a deteriorating humanitarian situation, violence against children and adolescents in the family and in the community is a serious issue. Emerging research indicates potential success of culturally-informed low-intensity psychological interventions. Yet, there remains a desperate need for further psychological research, support, assistance and interventions.
